# Genome-based characterization of *Escherichia coli* causing bloodstream infection through next-generation sequencing

**DOI:** 10.1371/journal.pone.0244358

**Published:** 2020-12-23

**Authors:** Rafika Indah Paramita, Erni Juwita Nelwan, Fadilah Fadilah, Editha Renesteen, Nelly Puspandari, Linda Erlina

**Affiliations:** 1 Department of Medical Chemistry, Faculty of Medicine, Universitas Indonesia, DKI Jakarta, Indonesia; 2 Bioinformatics Core Facilities—IMERI, Faculty of Medicine, Universitas Indonesia, DKI Jakarta, Indonesia; 3 Master’s Programme in Biomedical Sciences, Faculty of Medicine, Universitas Indonesia, DKI Jakarta, Indonesia; 4 Department of Internal Medicine, Faculty of Medicine, Universitas Indonesia, DKI Jakarta, Indonesia; 5 Infectious Disease and Immunology Research Center—IMERI, Faculty of Medicine, Universitas Indonesia, DKI Jakarta, Indonesia; 6 Centre for Research and Development of Biomedical and Basic Health Technology, National Institute of Health Research and Development, Ministry of Health, DKI Jakarta, Indonesia; Nitte University, INDIA

## Abstract

*Escherichia coli* are one of the commonest bacteria causing bloodstream infection (BSI). The aim of the research was to identify the serotypes, MLST (Multi Locus Sequence Type), virulence genes, and antimicrobial resistance of *E*. *coli* isolated from bloodstream infection hospitalized patients in Cipto Mangunkusumo National Hospital Jakarta. We used whole genome sequencing methods rather than the conventional one, to characterized the serotypes, MLST (Multi Locus Sequence Type), virulence genes, and antimicrobial resistance (AMR) of *E*. *coli*. The composition of *E*. *coli* sequence types (ST) was as follows: ST131 (*n* = 5), ST38 (*n* = 3), ST405 (*n* = 3), ST69 (*n* = 3), and other STs (ST1057, ST127, ST167, ST3033, ST349, ST40, ST58, ST6630). Enteroaggregative *E*. *coli* (EAEC) and Extra-intestinal pathogenic *E*. *coli* (ExPEC) groups were found dominant in our samples. Twenty isolates carried virulence genes for host cells adherence and 15 for genes that encourage *E*. *coli* immune evasion by enhancing survival in serum. ESBL-genes were present in 17 *E*. *coli* isolates. Other AMR genes also encoded resistance against aminoglycosides, quinolones, chloramphenicol, macrolides and trimethoprim. The phylogeny analysis showed that phylogroup D is dominated and followed by phylogroup B2. The *E*. *coli* isolated from 22 patients in Cipto Mangunkusumo National Hospital Jakarta showed high diversity in serotypes, sequence types, virulence genes, and AMR genes. Based on this finding, routinely screening all bacterial isolates in health care facilities can improve clinical significance. By using Whole Genome Sequencing for laboratory-based surveillance can be a valuable early warning system for emerging pathogens and resistance mechanisms.

## Introduction

There is currently enlarge in the prevalence of infections worldwide due to multidrug-resistant (MDR). Due to their correlation with a high level of mortality and morbidity which are triggered by the insufficient of potent antibiotics, Gram-negative bacteria became a critical threat to global public health [1–3]. The production of Extended-spectrum beta-lactamase (ESBL) became the most significant determinant of AMR that spreads rapidly among Enterobacteriaceae [4, 5].

*Escherichia coli* naturally inhabits the human gastrointestinal tract (GIT) and classified as Gram-negative commensals bacterium. Two main classification of pathogenic *E*. *coli—*diarrheagenic *E*. *coli* (DEC) and extra-intestinal pathogenic *E*. *coli* (ExPEC)—are perceived, varying in their associated clinical syndromes and virulence genes. Extraintestinal pathogenic *E*. *coli* (ExPEC) can cause a diversity of infections, including sepsis, neonatal meningitis, and urinary tract infections (UTI). The urinary tract is the most commonly infected by these bacteria, which then, is a common source for bloodstream infections [6]. Through horizontal transfer and other mechanisms, commensal *E*. *coli* regularly obtains pathogenicity, virulence and multi-drug resistance characteristics of the pathogenic *E*. *coli*. Virulent *E*. *coli* strains then share pathogenic, virulence, and resistance genes with avirulent or less virulent strains, allowing the appearance of pathogenesis beyond their natural characters [7].

There are six well-recognized pathotypes of DEC such as enterotoxigenic *E*. *coli* [ETEC], enteropathogenic *E*. *coli* [EPEC], enteroaggregative *E*. *coli* [EAEC], Shiga toxin (Stx)-producing *E*. *coli* [STEC], enteroinvasive *E*. *coli* [EIEC], and diffusely adherent *E*. *coli* [DAEC], are identified as the leading etiological agents of childhood and travellers’ diarrhoea. EAEC has long been considered as an intestinal pathogen and thereof unlikely to cause disease in normal patients outside of the intestinal tract [8]. However, current studies have related EAEC strains with fatal haemolytic uremic syndrome outbreaks and urinary tract infection [8, 9].

Whole genome sequencing (WGS) as a sophisticated molecular diagnostics have shown the emergence of *E*. *coli* strains that cause fatal diarrhoea due to the combination of the virulence genes [10]. The virulence genes are important to understand the pathogenic properties of the causative organisms. The virulence gene is an agent that forms itself in or within its host by increasing its able to trigger disease effectively and efficiently. The virulence genes in certain *E*. *coli* strains was associated with serious outbreaks in Denmark between 2014–2015 [11]; in Italia between 2016–2017, where it caused 7.1% 30-day mortality rate [12]; in China in 1999, where it caused 177 deaths [13]; and in Germany in 2011, where it killed 54 people [14]. Molecular genomic studies of human infections of *E*. *coli* in Indonesia, especially in Jakarta are unusual and no report is available regarding specific subtypes causing infections in Jakarta. The purpose of the study was to characterize the serotypes, MLST (Multi Locus Sequence Type), virulence genes, and antimicrobial resistance of *E*. *coli* isolated from bloodstream infection patients in Cipto Mangunkusumo National Hospital Jakarta.

## Methods

### DNA isolation and whole genome sequencing

From blood samples from bloodstream infection patients the *E*. *coli* were isolated and then it cultured in Lactose Broth medium in the laboratory facilities at the Centre for Research and Development of Biomedical and Basic Health Technology, National Institute of Health Research and Development, Ministry of Health, Indonesia, between January–December 2018. The DNA was isolated from cultured *E*. *coli u*sing a Geneaid Presto^TM^ Mini gDNA Bacteria Kit. All of the isolation and whole genome sequencing methods of 22 samples of *Escherichia coli* were described in Nelwan, *et al*, 2020 [15]. This research was performed in strict conformity with the suggestions in the guide of Helsinki Declaration and the research protocol was approved by Health Research Ethics Committee, National Institute of Health Research and Development (HREC-NIHRD) with protocol approval number: LB.02.01/KE.014/2019.

### Bioinformatics analysis

The paired-end reads from each *Escherichia coli* isolate were checked for the quality using FastQC 0.11.4 [16]. Before assembly, all reads with low quality were removed after quality control with Trimmomatic software [17]. Raw genome data have been submitted to the NCBI (https://www.ncbi.nlm.nih.gov/) and are available under BioProject accession number PRJNA596854 (https://www.ncbi.nlm.nih.gov/bioproject/PRJNA596854). According to this research aim, the analyses included: Multi-Locus Sequence Typing (MLST) determination using MLST 2.0.4 [18] (database version: 2.0.0 (2020-06-29)), serotyping using SeroTypeFinder 2.0.1 [19] (database version: 1.0.0 (2019-02-27)), virulence genes identification using VirulenceFinder 2.0 [20] (database version: 2020-05-29), and antimicrobial resistance (AMR) genes identification using ResFinder 3.2 [21] (database version: 2020-06-02). VirulenceFinder identified 29 virulence genes, of which 12 are ExPEC-associated, 16 are EAEC-associated, and 1 is EPEC-associated, irrespective of pathotype. For the detection standard parameters were set as follows: VirulenceFinder and SerotypeFinder, 85% sequence identity and 60% sequence coverage; ResFinder, 90% sequence identity and 60% sequence coverage; MLST, using the seven loci (gyrB, adk, icd, purA, recA, fumC, and mdh) scheme.

In this research, the 24 *E*. *coli* genomes were analyzed and compared with 26 diverse *E*. *coli* genomes (S1 Table) using PhaME pipelines [22] which applied a maximum likelihood phylogeny by RAxML v7.2.8, with the GTR model of nucleotide substitution, for model of rate heterogeneity we use the GAMMA, and 100 bootstrap replicates. The phylogeny was midpoint-rooted and diagramed using the interactive Tree of Life software (iTOL v.3).

## Results

### Multi-locus sequence typing and serotyping

From total of 22 *E*. *coli* isolates, 12 different STs were identified. Five (22,7%) *E*. *coli* samples are belonged to ST131 and all of this five had serotype O25:H4. Each of ST38, ST405, and ST69 were observed three (13,6%) out of 22 *E*. *coli* isolates. Of these three, two (66,7%) ST38 had serotype O86:H18, and another one had serotype unknown O-group but belonged to H30. All of the ST405 had serotype O102:H6 and the three of ST69 had different serotype, i.e O15:H6, O15:H18, O15:H1 (Table 1). Each of ST1057, ST127, ST167, ST3033, ST349, ST40, ST58, ST6630 were observed one (4,5%) out of 22 *E*. *coli* isolates. Strains with the same serotype and ST typically harboured the same set of virulence genes, such as O25:H4 (ST131) isolates, O102:H6 (ST405), and O86:H18 (ST38).

**Table 1:** Sequence types, serotypes, and virulence genes of 22 *E*. *coli* isolates.

Sample name	ST	Serotype	Phylogroup	Virulence Genes			
				Adhesins	Toxin	Siderophores	Protectins
**RSCM_EC_0203**	ST40	H21	B1	*lpfA*, *eae*		*iroN*	*iss*
**RSCM_EC_0709**	ST58	O8:H10	B1	*lpfA*			*iss*
**RSCM_EC_0507**	ST167	O89:H21	A				
**RSCM_EC_1316**	ST127	O6:H31	B2	*sfaS*	*cnf1*, *vat*, *senB*, *pic*	*iroN*	*iss*
**RSCM_EC_1114**	ST3033	O25:H31	B2		*vat*		
**RSCM_EC_0102**	ST1057	O154:H5	B2	*lpfA*	*cnf1*, *ireA*, *sat*, *vat*	*ireA*, *iroN*	
**RSCM_EC_0406**	ST131	O25:H4	B2	*iha*	*sat*, *senB*		*iss*
**RSCM_EC_0911**	ST131	O25:H4	B2	*iha*	*cnf1*, *sat*		*iss*
**RSCM_EC_2442**	ST131	O25:H4	B2	*iha*	*sat*, *senB*		*iss*
**RSCM_EC_2137**	ST131	O25:H4	B2	*iha*	*cnf1*, *sat*, *senB*		*iss*
**RSCM_EC_1833**	ST131	O25:H4	B2	*nfaE*	*pic*		*iss*
**RSCM_EC_0608**	ST6630	O1:H25	F	*lpfA*, *air*, *eilA*			
**RSCM_EC_2240**	ST349	O86:H2	D	*eilA*		*iroN*	*iss*
**RSCM_EC_2036**	ST405	O102:H6	D	*air*, *eilA*			
**RSCM_EC_1526**	ST405	O102:H6	D	*air*, *eilA*			
**RSCM_EC_1013**	ST405	O102:H6	D	*air*, *eilA*			
**RSCM_EC_0305**	ST38	O86:H18	D	*aap*, *air*, *eilA*, *nfaE*	*astA*		*iss*
**RSCM_EC_1628**	ST38	O86:H18	D	*aap*, *air*, *eilA*, *nfaE*	*astA*		*iss*
**RSCM_EC_1418**	ST38	H30	D	*aap*, *air*, *eilA*, *iha*, *aatA*, *agg3B*, *agg3C*, *agg3D*, *agg5A*, *aggR*	*sat*		*iss*
**RSCM_EC_2341**	ST69	O15:H1	D	*lpfA*, *air*, *eilA*	*senB*		*iss*
**RSCM_EC_1732**	ST69	O15:H6	D	*lpfA*, *air*, *eilA*	*tsh*	*iroN*	*iss*
**RSCM_EC_1935**	ST69	O15:H18	D	*lpfA*, *iha*, *aap*, *air*, *eilA*, *aatA*, *aar*, *aggA*, *aggB*, *aggC*, *aggD*, *aggR*	*sat*		*iss*

### Distribution of virulence genes

Table 1 as well display the classification of virulence genes among the sequenced *E*. *coli* isolates. The virulence genes distribution in pathotypes species of *E*. *coli* were described in Table 2. A total of 20 (20,9%) *E*. *coli* isolates carried virulence genes for host cells adherence. These contained 11 (55%) for *eilA*, 10 (50%) for *air*, 7 (35%) for *lpfA*, 6 (30%) for *iha*, 3 (15%) for each *nfaE* and *aap*, 2 (10%) for each *aggR* and *aatA*, 1 (5%) for each *aar*, *sfaS* and *eae*. A total of 14 *E*. *coli* isolates had virulence genes that promote toxin production. The composition of virulence genes that promote toxin was 4 (28,6%) for *cnf1*, 6 (42,8%) for *sat*, 3 (21,4%) for *vat*, 5 (35,7%) for *senB*, 2 (14,3%) for each *pic* and *astA*, 1 (7,1%) for each *ireA* and *tsh*. A total of 5 *E*. *coli* isolates had virulence genes that produce iron acquisition systems (siderophores). Of these five, 5 (100%) for *iroN* and 1 (20%) for *ireA*. A total of 15 (68,2%) *E*. *coli* isolates had *iss*, a component that promotes *E*. *coli* immune evasion by increasing serum survival.

**Table 2:** Virulence genes distribution in *E*. *coli*.

Gene	Gene description	Pathotype Species	References
*aggR*	Transcriptional activator	EAEC	[23]
*aar*	AggR-activated regulator	EAEC	[23]
*aap*	Dispersin, antiaggregation protein	EAEC	[23]
*aatA*	Dispersin transporter protein	EAEC	[23]
*aggA*	AAF/I fimbrial subunit	EAEC	[23]
*aggB*	AAF/I fimbrial subunit	EAEC	[23]
*aggC*	AAF/I fimbrial subunit	EAEC	[23]
*aggD*	AAF/I fimbrial subunit	EAEC	[23]
*agg3B*	AAF/III fimbrial subunit	EAEC	[23]
*agg3C*	AAF/III fimbrial subunit	EAEC	[23]
*agg3D*	AAF/III fimbrial subunit	EAEC	[23]
*agg5A*	AAF/V fimbrial subunit	EAEC	[23]
*air*	Enteroaggregative immunoglobulin repeat protein	EAEC	[23]
*astA*	Heat-stable enterotoxin 1	EAEC	[23]
*cnf1*	cytotoxic necrotizing factor 1	ExPEC	[23]
*eilA*	Salmonella HilA homolog	EAEC	[23]
*eae*	intimin	EPEC	[24]
*iha*	Adherence protein	ExPEC	[25]
*ireA*	Siderophore receptor	ExPEC	[23]
*iroN*	Salmochelin receptor	ExPEC	[25]
*iss*	Increased serum survival	ExPEC	[23]
*lpfA*	Long polar fimbriae	EAEC	[23]
*pic*	Serin protease autotransporter	ExPEC	[25]
*nfaE*	Diffuse adherence fibrillar adhesin	ExPEC	[26]
*sfaS*	S-fimbriae	ExPEC	[23]
*sat*	Secreted autotransporter toxin	ExPEC	[25]
*senB*	Plasmid encoded enterotoxin	ExPEC	[27]
*tsh*	Temperature-sensitive hemagglutinin	ExPEC	[28]
*vat*	Vacuolating autotransporter toxin	ExPEC	[28]

Isolates that harboured the virulence genes of EAEC were characteristically typical EAEC by genomic criteria. The EAEC showed highly heterogeneous with the existence of a substantial number of genes typically correlated with other *E*. *coli* pathotypes, i.e. extraintestinal pathogenic *E*. *coli* (ExPEC) and EPEC. ExPEC genes found among the EAEC strains included i) increased serum survival-encoding gene, iss (63.6%); ii) Secreted autotransporter toxin, *sat* (18.2%); iii) Plasmid encoded enterotoxin, *senB* (9.1%); iv) Temperature-sensitive hemagglutinin, *tsh* (9.1%), and Salmochelin receptor, *iroN* (18.2%). *LpfA* was the only virulence gene to differ in frequency between the phylogenetic groups, as it was found in all B1 isolates, phylogroup B2, phylogroup F, and phylogroup D.

### Antimicrobial resistance

A total of 22 (100%) *E*. *coli* carried genes encoding macrolide resistance enzymes (Table 3). Those include 22 (100%) of *mdf(A)*, 9 (40.9%) of *mph(A)*, 1 (4.5%) for *ere(B)*, 1 (4.5%) of *erm(B)*, and 1 (4.5%) for *Inu(F)*. A total of 19 (86.4%) *E*. *coli* isolates showed anti beta-lactam resistance genes. The proportions of genes encoding the beta-lactam enzymes are blaCTX-M-15 in 6 (27.3%) isolates, *blaCTX-M-27* in 5 (22.7%) isolates, *blaTEM-1B* in 8 (36.4%) isolates, *blaDHA-1* in 3 (13.6%) isolates, *blaOXA-10* in 1 (4.5%) isolate, *blaOXA-1* in 3 (13.6%) isolates, *blaCTX-M-55* in 2 (9.1%) isolates, *blaCARB-2* in 1 (4.5%) isolate and *blaSHV-120* in 1 (4.5%) isolate. The overall proportion of *dfrA* genes encoding trimethoprim resistance enzymes was 12 (54.5%) isolates. The composition of *dfrA17* was 5 (22.7%) isolates, followed by 3 (13.6%) for *dfrA14*, 2 (9.1%) for *dfrA12* and 1 (4.5%) for each *dfrA1*, *dfrA7*, and *dfrA16* genes. The compositions of genes encoding chloramphenicol resistance enzymes were 3 (13.6%) for *catB3*, 3 (13.6%) for *catA1*, 3 (13.6%) for *floR* and 1 (4.5%) for *catA2*. A total of 15 (63.6%) isolates carried genes against aminoglycoside. The gene *aadA5* was detected in 4 (18.2%), *aph(6)-Id* in 8 (36.4%), *aac(3)-IId* in 5 (22.7%), *aac(6')-Ib-cr* in 3 (13.6%), *aadA2* in 2 (9.1%), each *aadA1* and *aph(3”)-Ib* in 2 (4.5%), *aph(6)-Id* in 1(4.5%), *Aac(6”)-Iai* in 1(4.5%), *aac(3)-IIa* in 3 (13.6%), and *aadA2* in 2 (9.1%) *E*. *coli* isolates.

**Table 3:** Antimicrobial resistance genes.

Sample_name	Antibiotics (Identity%; Query/Template length)					
	Aminoglycoside	Macrolide	Quinolones	Beta-Lactam	Phenicol	Trimethoprim
**RSCM_EC_0203**		*mdf(A)* (99.11; 1233/1233)				
**RSCM_EC_0709**	*aac(3)-IId* (99.88; 861/861)	*mdf(A)* (98.46; 1233/1233)	*qnrS1* (100; 657/657)	*blaCTX-M-55* (100; 876/876)		
**RSCM_EC_0507**	*aac(3)-IId* (99.88; 861/861) *aadA2* (100; 792/792)	*mdf(A)* (99.92; 1233/1233) *mph(A)* (100; 906/906)	*qepA4* (100; 1536/1536)	*blaCTX-M-15* (100; 876/876) *blaTEM-1B* (100; 861/861)	*catA1* (99.85; 660/660)	*dfrA12* (100; 498/498)
**RSCM_EC_1316**		*mdf(A)* (97.89; 1233/1233)		*blaCTX-M-15* (100; 876/876)		
**RSCM_EC_1114**		*mdf(A)* (97.89; 1233/1233)				
**RSCM_EC_0102**	*Aph(3”)-Ib* (100; 804/804) *aph(6)-Id* (100; 837/837)	*mdf(A)* (97.89; 1233/1233)		*blaTEM-1B* (100; 837/837)		*dfrA7* (100; 474/474)
**RSCM_EC_0406**	*aac(3)-IId* (99.88; 861/861) *Aac(6”)-Iai* (100; 567/567)	*mdf(A)* (97.81; 1233/1233) *mph(A)* (100; 906/906)	*qnrB4* (100; 645/645)	*blaDHA-1* (100; 1140/1140) *blaOXA-10* (100; 637/801) *blaSHV-120*, (100; 861/861) *blaTEM-1B* (100; 861/861)		*dfrA16* (100; 474/474) *dfrA17* (100; 474/474)
**RSCM_EC_0911**	*aac(3)-IIa* (99.77; 861/861) *aac(6')-Ib-cr* (100; 600/600) *aadA5* (100; 789/789)	*mdf(A)* (97.81; 1233/1233) *mph(A)* (99.58; 720/921)	*aac(6')-Ib-cr* (100; 600/600)	*blaCTX-M-15* (100; 876/876) *blaOXA-1* (100; 831/831)	*catB3* (100; 442/633)	*dfrA17* (100; 474/474)
**RSCM_EC_2442**		*mdf(A)* (97.81; 1233/1233)		*blaCTX-M-27* (100; 876/876)		
**RSCM_EC_2137**	*aac(6')-Ib-cr* (100; 600/600) *aac(3)-IIa* (99.77; 861/861)	*mdf(A)* (97.81; 1233/1233)	*aac(6')-Ib-cr* (100; 600/600)	*blaCTX-M-15* (100; 876/876) *blaOXA-1* (100; 831/831)	*catB3* (100; 442/633)	
**RSCM_EC_1833**	*aac(3)-IId* (99.88; 861/861) *aac(6')-Ib-cr* (100; 600/600) *aadA2* (100; 565/792)	*mdf(A)* (97.81; 1233/1233) *mph(A)* (99.58; 720/921) *ere(B)* (100; 1189/1260)	*aac(6')-Ib-cr* (100; 600/600)	*blaCARB-2* (100; 867/867) *blaCTX-M-15* (100; 876/876) *blaTEM-1B* (100; 861/861) *blaOXA-1* (100; 831/831)	*catA1* (99.85; 660/660) *catA2* (96.11; 642/642) *catB3* (100; 442/633)	*dfrA12* (100; 498/498) *dfrA14* (100; 474/474)
**RSCM_EC_0608**		*mdf(A)* (98.46; 1233/1233)				
**RSCM_EC_2240**	*aadA1* (99.87; 792/792)	*mdf(A)* (98.05; 1233/1233) *mph(A)* (100; 906/906)	*qnrB4* (100; 645/645)	*blaDHA-1* (100; 1140/1140)		
**RSCM_EC_2036**	*aph(6)-Id* (100; 837/837) *aph(3'')-Ib* (100; 804/804)	*mdf(A)* (97.97; 1233/1233)	*qepA1* (100; 1536/1536)	*blaCTX-M-27* (100; 876/876)	*floR* (98.19; 1214/1215)	
**RSCM_EC_1526**	*aph(6)-Id* (100; 698/831) *aadA5* (100; 789/789) *aph(3'')-Ib* (100; 804/804)	*mdf(A)* (97.97; 1233/1233) *mph(A)* (100; 906/906)	*qepA1* (99.61; 1542/1536)	*blaCTX-M-27* (100; 876/876)	*floR* (98.19; 1214/1215)	*dfrA17* (100; 474/474)
**RSCM_EC_1013**	*aac(3)-IIa* (99.77; 861/861) *aph(3'')-Ib* (100; 804/804) *aph(6)-Id* (100; 837/837) *aadA5* (100; 789/789)	*mdf(A)* (97.97; 1233/1233) *mph(A)* (100; 906/906) *erm(B)* (99.73; 738/738)		*blaCTX-M-15* (100; 876/876)	*floR* (98.19; 1214/1215)	*dfrA17* (100; 474/474)
**RSCM_EC_0305**	*aph(6)-Id* (100; 831/831) *Aph(3”)-Ib* (100; 804/804) *aadA5* (100; 789/789)	*mdf(A)* (98.13; 1233/1233) *mph(A)* (100; 906/906)		*blaCTX-M-27* (100; 876/876)		*dfrA17* (100; 474/474)
**RSCM_EC_1628**		*mdf(A)* (98.13; 1233/1233)		*blaCTX-M-27* (100; 876/876)		
**RSCM_EC_1418**	*aadA1* (100; 789/789) *aph(3'')-Ib* (100; 803/804) *aph(6)-Id* (100; 837/837)	*mdf(A)* (98.13; 1233/1233)		*blaTEM-1B* (100; 861/861)	*catA1* (99.55; 660/660)	*dfrA1* (100; 474/474)
**RSCM_EC_2341**		*mdf(A)* (97.57; 1233/1233)	*qnrB4* (100; 645/645)	*blaTEM-1B* (100; 861/861) *blaDHA-1* (100; 1140/1140)		*dfrA17* (100; 474/474)
**RSCM_EC_1732**	*aadA17* (98.86; 792/792) *aph(3'')-Ib* (100; 804/804) *aac(3)-IId* (99.88; 861/861) *aph(6)-Id* (100; 837/837)	*mdf(A)* (97.57; 1233/1233) *mph(A)* (100; 906/906) *Inu(F)* (100; 822/822)	*qnrS1* (100; 657/657)	*blaCTX-M-55* (100; 876/876) *blaTEM-1B* (100; 861/861)		*dfrA14* (100; 474/474)
**RSCM_EC_1935**	*aph(6)-Id* (100; 837/837) *aph(3'')-Ib* (100; 529/804)	*mdf(A)* (97.57; 1233/1233)		*blaTEM-1B* (100; 861/861)		*dfrA14* (100; 474/474)

Ten of *E*. *coli* isolates were predicted to be resistant to ciprofloxacin (Table 4) due to amino acid substitutions in quinolone-resistance-determining-regions (QRDR) of *parC* gene (S80I, E84V) and/or QRDR of *gyrA* gene (S83L, D87N). Moreover, several SNPs resulting in amino acid substitutions were also defined in *parE* gene (I529L, S458A). From this result, *E*. *coli* isolates that show Ciprofloxacin isolates were ST131, ST405, and ST167.

**Table 4:** Amino acid substitutions related to ciprofloxacin predicted-resistance using ResFinder 2.1.

Sample_name	Amino acid changes		
	*gyrA*	*parC*	*parE*
**RSCM_EC_0406**	S83L, D87N	S80I, E84V	I529L
**RSCM_EC_0911**	S83L, D87N	S80I, E84V	I529L
**RSCM_EC_1833**	S83L	-	I529L
**RSCM_EC_2137**	S83L, D87N	S80I, E84V	I529L
**RSCM_EC_2442**	S83L, D87N	S80I, E84V	I529L
**RSCM_EC_0507**	S83L, D87N	S80I	S458A
**RSCM_EC_1013**	S83L, D87N	S80I	S458A
**RSCM_EC_1526**	S83L, D87N	S80I	S458A
**RSCM_EC_2036**	S83L, D87N	S80I	S458A

### Phylogenomic analysis of the *E*. *coli* isolates

**Fig 1:**
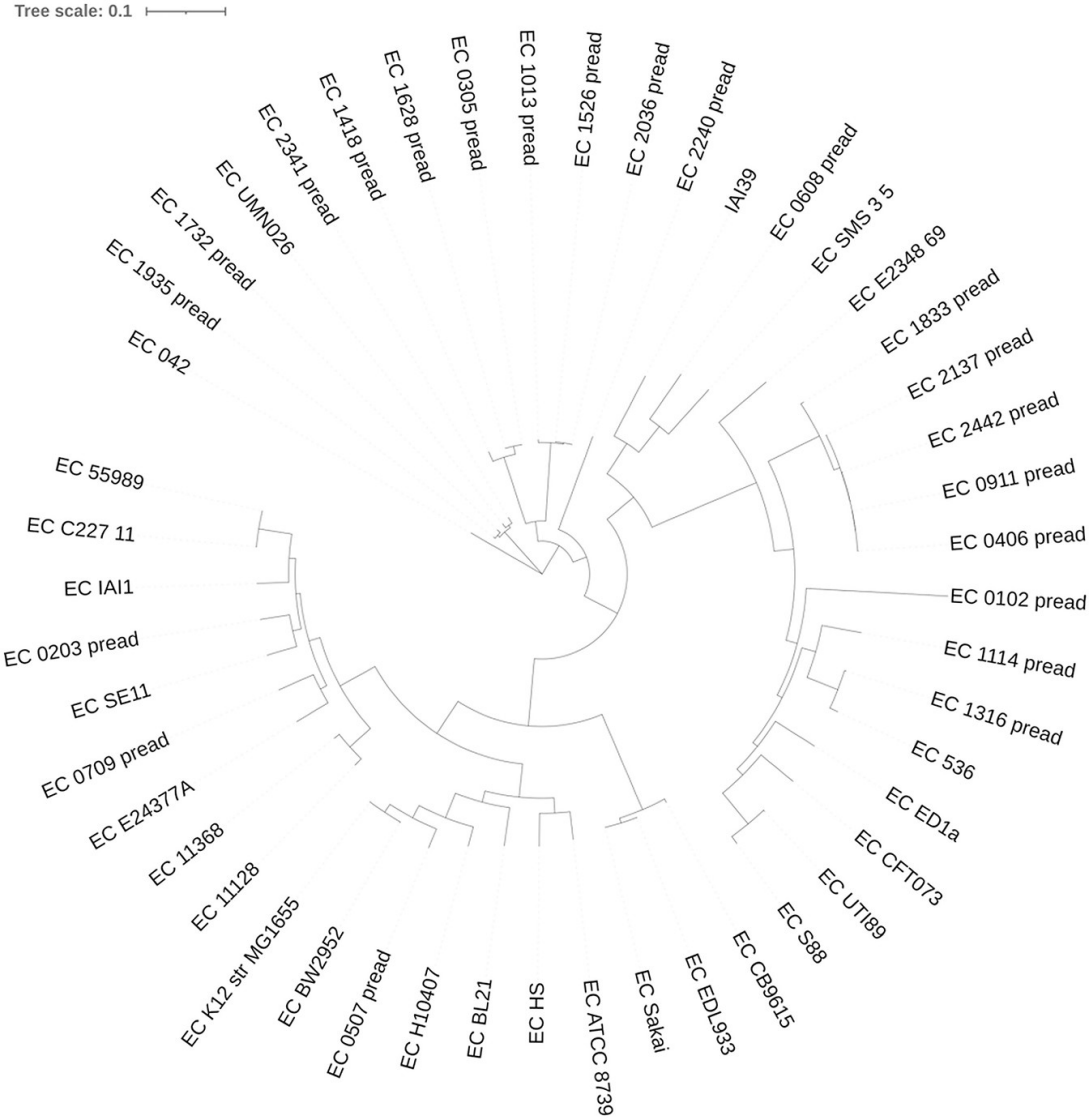
Phylogenetic tree of the E. coli isolates and 26 E. coli references.

In general, *E*. *coli* may be distributed into 7 phylogenetic categories as follows: A, B1, B2, C, D, E, and F [29]. Based on the phylogenetic tree of the E. coli isolates and 26 E. coli references as shown in Fig 1, eleven strains of the sequenced EAEC strains owned to the phylogroup D, and one strain of phylogroup F. Among phylogroup D, there were four subgroups, which showed in lineages 1–4. Strain ST349 was the only strain in lineage 1; lineage 2 strains included O102:H6 (ST405); lineage 3 strains included ST38; lineage 4 strains included ST69. Among phylogroup B2, there was also 4 lineage. In lineage 1, H31 isolate was included; O154:H5 (ST1057) isolate which is the only strain available, included in lineage 2; lineage 3 strains included four of O25:H4 (ST131); one of the O25:H4 (ST131) isolate was included in lineage 4 because it had different virulence genes than the other four (Fig 1).

## Discussion

This research showed a huge variation of *E*. *coli* strains prevalent in Cipto Mangunkusumo hospital as assessed by Multi-Locus Sequence Typing (MLST) and Serotyping. Meanwhile, strains O25:H4-ST131 was found to predominate among other 11 ST: ST38, ST405, ST69, ST1057, ST127, ST167, ST3033, ST349, ST40, ST58, ST6630. Each of ST38, ST405, and ST69 was found in three isolates. But for each ST38 and ST69 have different serotypes within it. These results are identical to the results from other regions that described the distribution and prevalence of these endemic clones in health facilities [30–32]. The variation of clones discovered in the hospital might propose sporadic introductions of various strains to the hospital from the community. To investigate whether identical STs are clonal associated, SNP differences among isolates and phylogenetic assessment proposed the presence of multiple clones of *E*. *coli* in these settings.

Generally, the levels of antimicrobial resistance in *E*. *coli* isolates were inspected to be elevated. Based on assessment of resistance genes, 22 of the *E*. *coli* isolates carried genes encoding Macrolide, with *mdf(A)* being prevalent (100% appeared in 22 isolates) followed by *mph(A)*, *ere(B)*, *erm(B)*, and *Inu(F)*. Gene *mdfA* encodes a putative membrane protein (*mdfA*) of 410 amino acid residues which are the main facilitators of the transport protein superfamily. Cells that express *mdfA* from multicopy plasmids are considerably more resistant to various groups of zwitterionic lipophilic or cationic compounds such as benzalkonium, rhodamine, tetracycline, daunomycin, ethidium bromide, puromycin, rifampin, and tetraphenylphosphonium. Although, *mdfA* also provides resistance to chemically unrelated, clinically essential antibiotics such as fluoroquinolones, erythromycin, chloramphenicol, and aminoglycosides [33]. Gene *mph(A)* conferring resistance to macrolides including azithromycin and erythromycin also was discovered in a high quantity. The appearance of the *mph(A)* in *E*. *coli* isolates of the current research was 40.9%, and thus, higher than the 13% that was identified in *E*. *coli* from 5 countries from 4 continents by Nguyen *et al* [34]. and lower than the 50% that was found in *E*. *coli* from tertiary care hospital in Moshi, Tanzania by Sonda *et al* [7]. High risks to azithromycin and erythromycin can be one of the feasible reasons that allow the emergence of resistance to macrolides [35].

In this study, *blaTEM-1B* being predominant among other genes encoding beta-lactamase and followed by blaCTX-M-15 and *blaCTX-M-27*. In a tertiary hospital in Dar es Salaam, Manyahi et al [36] found blaCTX-M-15 as the most predominant gene (90.6%) and Sonda et al. [7] who found *blaOXA-1* as the most predominant gene in a tertiary hospital in Moshi, Tanzania. In the previous research, O25:H4-ST131 was mentioned to be a main reason of MDR *E*. *coli* infections [7, 37, 38]. The presence of *aac(6’)Ib-cr* encodes low ciprofloxacin resistance by itself (as well as aminoglycoside resistance), and generally requires further mutations (e.g. in the *gyrA* or *parC* chromosomes) to provide high levels of resistance. Other research in the Korea [32], US [39], and Brazil [40] have confirmed identical results with this research of the co-carriage of *aac(6’)Ib-cr*, ESBL genes and the presence of ciprofloxacin resistance in *E*. *coli* ST131. This plausibly clarify the discovered relationship between transport of *aac(6’)Ib-cr* and CTX-M ESBL and ciprofloxacin resistance [41].

This current research also characterized virulence genes in all *E*. *coli* isolates. Based on our results, we found the specific virulence genes in ExPEC and EAEC groups. Adherence protein (*iha*) was found dominating in ExPEC adherence virulence genes. We also found several toxins which belong to ExPEC group, such *as cnf1*, *ireA*, *sat*, *vat*, *senB*, *pic*, and *tsh*. Another prevalent group of virulence gene were liable for *E*. *coli* immune evasion by increasing serum survival (*iss*). The *iss* gene recognized for its role in ExPEC for enhanced survival of bacteria in the serum. The *iss* gene is located on plasmid ColV, a huge virulence plasmid typical of avian pathogenic *E*. *coli* strains, which reveal that a replacement of plasmids and, as the result, replacement of those virulence genes is feasible between human and avian pathogenic *E*. *coli* strains [25].

EAEC-specific virulence genes present in our 12 isolates included aggregative adherence fimbria (AAF) variant I; AggR, a global regulator of EAEC virulence; AggR-activated regulator, *aar;* dispersin, required for proper dispersal of AAFs on the bacterial surface; and the *aat* transporter system, which mediates dispersin secretion [42]. The *aggR* gene was found in ST38 (H30) sample and both *aggR* and *aar*, was found in ST69 (O15:H18) sample. Strains harbouring the *aggR* regulon or its constituents have been termed typical EAEC. We also found two samples that harbouring EAEC toxin, i.e. *astA*, which both of them belong to ST38 (O86:H18). These virulence genes have been defined as suggestive of virulent serotypes and may be used as reliable markers for the detection of pathogenic *E*. *coli* [43].

Based on our results, the existence of a substantial number of genes characteristically correlated with another *E*. *coli* pathotypes, such extraintestinal pathogenic *E*. *coli* (ExPEC). ExPEC genes found among the EAEC strains included i) increased serum survival-encoding gene, iss (63.6%); ii) Secreted autotransporter toxin, *sat* (18.2%); iii) Plasmid encoded enterotoxin, *senB* (9.1%); iv) Temperature-sensitive hemagglutinin, *tsh* (9.1%), and Salmochelin receptor, *iroN* (18.2%). The similar result was also found in another study by Boisen *et*. *al*. 2020 [23], that showed ten ExPEC genes presented in EAEC strains. These finding showed that related to virulence genes, EAEC strains are highly heterogeneous.

Potential clinical implications for the results received are that caution must be taken when explaining and utilizing microbiology outcomes. A study from Havt *et al* [44], showed that malnutrition status of children aged 6–24 months was related to virulence genes of *aatA* and *aar*. This research underscore that *E*. *coli* should not be considered as non-pathogenic until the determinants of pathogenic and antimicrobial resistance have been verified to be missing. In addition, the WGS-based result suggested that there are nosocomial transmissions in the hospital, thus encouraging the formulation of pragmatic antimicrobial management, control initiatives and infection prevention.

We understand and acknowledge the limitations of this study. The assessment was carried out on a limited number of *E*. *coli* isolates, which may limit generalization of the results. With a limited number of the isolates analyzed, it is essential to figure out that another limitation that this work may suffer is the insufficiency of deeper statistical analysis to associate the isolates resistance and virulence results with patient characteristics such as room, age, ward, and gender. Also, the presence of genes that encode various resistance and virulence genes only shows the genes that are in the isolates.

## Conclusions

The *E*. *coli* isolated from 22 patients in Cipto Mangunkusumo National Hospital Jakarta showed high diversity in serotypes, sequence types, virulence genes, and AMR genes. Based on this finding, routinely screening all bacterial isolates in health care facilities can improve clinical significance. By using Whole Genome Sequencing for laboratory-based surveillance can be a valuable early warning system for emerging pathogens and resistance mechanisms.

## Supporting information

S1 Table *E. coli* reference genomes included in the phylogenomic analysis. (XLSX)
